# Prognostic Value of Peroxiredoxin-1 Expression in Patients with Solid Tumors: a Meta-Analysis of Cohort Study

**DOI:** 10.1155/2021/9508702

**Published:** 2021-03-03

**Authors:** Lianghe Jiao, Jing Wei, Jun Ye, Chuanmeng Zhang

**Affiliations:** ^1^Department of Thyroid and Breast Surgery, Taizhou People's Hospital, Taizhou, 225300 Jiangsu Province, China; ^2^Department of Obstetrics and Gynecology, Taizhou People's Hospital, Taizhou, 225300 Jiangsu Province, China; ^3^The Center for Translational Medicine, Taizhou People's Hospital, Taizhou, 225300 Jiangsu Province, China

## Abstract

**Methods:**

We comprehensively searched electronic databases, namely, PubMed, Web of Science, EMBASE, Chinese National Knowledge Infrastructure (CNKI), and WanFang databases up to December 2019. Hazard ratios (HRs) with 95% confidence intervals (CIs) were calculated to evaluate the association between PRDX1 protein expression and the survival of patients with solid tumors. Odds ratios (ORs) with 95% CIs were pooled to estimate the correlation between PRDX1 protein expression and clinicopathologic characteristics in the patients.

**Results:**

Seventeen cohort studies that involved 2,858 patients were included in this meta-analysis. The pooled results indicated that positive PRDX1 expression was related to poor overall survival (HR = 1.68, 95% CI: 1.24-2.27, *P* = 0.001) and disease-free survival (HR = 1.88, 95% CI: 1.31-2.70, *P* = 0.001). In addition, high PRDX1 expression was associated with large tumor size (OR = 1.69, 95% CI: 1.07-2.68, *P* = 0.025), advanced TNM stage (OR = 2.26, 95% CI: 1.24-4.13, *P* = 0.008), and poor tumor differentiation (OR = 0.59, 95% CI: 0.44-0.81, *P* = 0.001).

**Conclusions:**

PRDX1 overexpression is associated with poor outcomes of cancers and may serve as a prognostic biomarker for malignant patients. Hence, PRDX1 could be a new target for antitumor therapy.

## 1. Introduction

Cancer is one of the leading contributors to morbidity and mortality, with approximately 1,735,350 new cancer cases and 609,640 cancer-related deaths reported in the United States in 2018 [1]. Despite great advances in early detection and comprehensive treatment in recent years, most cancers still have poor prognosis [2, 3]. Early diagnosis and treatment monitoring can improve the prognosis of patients with cancer [4]. However, most cancer biomarkers lack sensitivity and specificity [5]. Therefore, identifying novel prognostic biomarkers is urgently needed not only for predicting outcomes but also for providing therapeutic targets for patients with cancer.

Reactive oxygen species (ROS) is involved in various physiological and pathological reactions in mammalian cells and plays a critical role in some diseases, including certain cancers [6, 7]. Peroxiredoxins (PRDXs) are a family of redox regulating proteins that could reduce ROS and maintain the stability of hydrogen peroxide in cells [8]. Meanwhile, these proteins can also inhibit ROS-induced tumor cell apoptosis and promote tumor cell survival. The mammalian PRDX family consists of six members (PRDX1 to PRDX6), which can be divided into three subgroups according to the number of cysteine residues: typical 2-cysteine (PRDX1-4), atypical 2-cysteine (PRDX5), and 1-cysteine (PRDX6) proteins [9]. Recent studies have reported that PRDXs are upregulated in various types of cancer and closely related to tumor progression, recurrence, and prognosis [10–14]. Thus, PRDXs have been identified as potential predictive and therapeutic biomarkers for cancer.

Peroxiredoxin-1 (PRDX1) is an affiliate of the PRDX antioxidant protein superfamily [15]. PRDX1 activity is implicated in several biological processes, such as cell differentiation, proliferation, and apoptosis [16]. In the nucleus, PRDX1 influences biological activities of gene regulation by associating with various transcription factors, including p53, androgen receptor (AR), and nuclear factor kappa B (NF-*κ*B), and then induces or inhibits cell death [8, 17, 18]. In the cytoplasm, PRDX1 shows antiapoptotic potential by interacting with an ROS-dependent signaling pathway [19]. An increasing number of studies have reported the association of high PRDX1 protein expression level with poor prognosis in patients with solid tumors. However, several publications revealed that the correlation is nonsignificant or even reversed. Thus, the real value of PRDX1 in predicting the prognosis of solid tumors remains controversial. In this regard, a meta-analysis is needed to determine the prognostic value of PRDX1 protein expression in patients with solid tumors.

## 2. Materials and Methods

### 2.1. Search Strategy

We performed this meta-analysis in accordance with the guidelines of the Preferred Reporting Items for Systematic Reviews and Meta-Analyses. The PubMed, Web of Science, EMBASE, Chinese National Knowledge Infrastructure (CNKI), and WanFang databases (up to November 2019) were systematically searched with the following keywords: (“peroxiredoxin-1” or “prdx1” or “prx1”), and (“tumor” or “cancer” or “carcinoma” or “neoplasm”), and (“prognosis” or “outcome” or “survival”). Manual searches were also conducted on the reference lists of the original articles to identify additional studies. Full-text articles published in English or Chinese were included.

### 2.2. Inclusion and Exclusion Criteria

Studies were enrolled according to the following criteria: (1) solid tumors were diagnosed by histopathology; (2) studies assessed the relationship between PRDX1 and prognostic outcomes, including overall survival (OS) or disease-free survival (DFS); (3) PRDX1 protein expression was detected in cancer tissues by immunohistochemistry stain or reverse phase protein array (RPPA) analysis and categorized into “high” and “low” groups; and (4) hazard ratios (HRs) with corresponding 95% confidence intervals (95% CIs) can be extracted directly or estimated with sufficient information. Articles were excluded according to the following criteria: (1) letters, reviews, abstracts, case reports, editorials, expert opinions, or animal experiments; (2) studies without sufficient information for estimating HRs with corresponding 95% CIs; (3) participants were not divided into two groups according to PRDX1 expression; and (4) studies with a sample size of less than 50.

### 2.3. Data Extraction and Quality Assessment

Data from each study were evaluated and extracted independently by two investigators (JLH and WJ). Any disagreement was resolved by a consensus with a third investigator (ZCM). The following information was collected in this meta-analysis: first author's name, publication year, country, cancer type, TNM stage, sample size, follow-up time, OS and DFS, and HR estimation with 95% CI. In studies that reported univariate and multivariate HR estimations, only the latter was selected because it considers confounding factors and is more accurate.

The quality of eligible studies was assessed independently by two authors (JLH and WJ) according to the Newcastle-Ottawa Scale (NOS). Any disagreement was resolved through discussion with another investigator (YJ). Each study was assigned an overall score ranging from 0 to 9 based on quality of selection, comparability, exposure, and outcome assessment. Investigations with scores higher than or equal to 6 were regarded as high-quality research.

### 2.4. Statistical analysis

The Stata 12.0 (STATA Corp., College Station, TX) software was used for all statistical analyses. The combined HRs and their 95% CIs were calculated to evaluate the relationship between PRDX1 expression and patient survival. For the overall results, HR and 95% CI greater than 1 indicated poor prognosis in patients with PRDX1 overexpression. Moreover, pooled odds ratio (OR) and their 95% CI were applied to assess the association between PRDX1 expression and the clinicopathological parameters of solid tumors. Heterogeneity among individual studies was analyzed using chi-squared *Q* test and *I*-squared statistical test. When the results (*I*^2^ > 50% or *P* < 0.05) indicated heterogeneity, the random effects model was applied for the meta-analysis; otherwise, the fixed effects model was adapted. Metaregression and subgroup analyses were conducted on studies sorted into subgroups according to similar variables. Sensitivity analysis was performed to verify the stability of the synthesis results by sequentially omitting each individual study. Publication bias was also statistically evaluated using Egger's test and visually assessed with a funnel plot. In case of significant publication bias, the trim-and-fill method was applied to validate the robustness of the summary results.

## 3. Results

### 3.1. Literature Search and Included Studies

**Figure 1:**
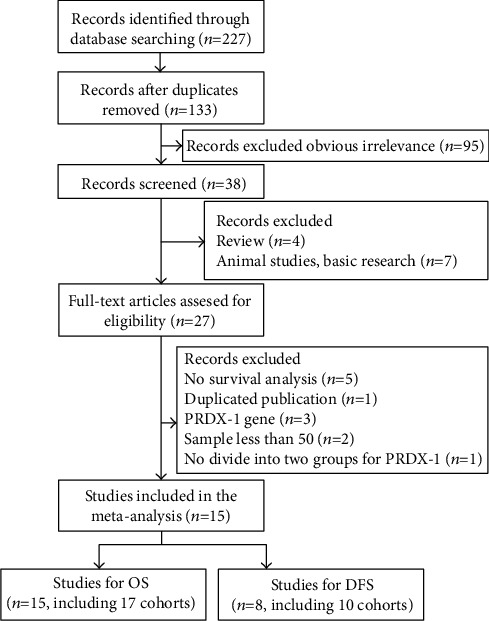
Flow diagram of the study selection process and specific reasons for exclusion in the meta-analysis.

A total of 227 records were retrieved from the PubMed, Web of Science, EMBASE, CNKI, and WanFang databases by using the above mentioned search strategy; of which, 94 studies were excluded because of duplicate records. And then 95 articles were removed because of obvious irrelevance. After screening the titles and abstracts, 27 papers were identified for full-text review. Finally, 15 articles met the inclusion criteria and were included in the meta-analysis. The study selection process is shown in Figure 1.

The basic characteristics of each article are summarized in Table 1. Among the 15 articles, 17 cohorts involving 2,858 patients were included, with sample sizes ranging from 55 to 712. All the included articles were cohort studies published between 2007 and 2019. These studies included patients from various regions who were diagnosed with 11 different types of cancer: ovarian cancer (OC) [8, 12], osteosarcoma (OSC) [20], colorectal cancer (CRC) [21], gastric cancer (GC) [22, 23], cholangiocarcinoma (CCA) [24, 25], esophageal squamous cell carcinoma (ESCC) [26, 27], pancreatic cancer (PC) [28], breast cancer (BC) [29], hepatocellular carcinoma (HCC) [30], gallbladder cancer (GBC) [31], and non-small cell lung cancer (NCSLC) [32]. All 17 cohorts reported data on OS, and 10 cohorts presented data on DFS. HRs and their 95% CIs obtained through COX multivariate analysis were directly reported in 14 cohorts, while the other data were calculated from the Kaplan-Meier curves. According to the NOS, every study gained score ≥ 6, indicating the high level of the methodological quality of all enrolled studies.

### 3.2. Correlation between PRDX1 and Clinicopathological Features

To analyze the role of PRDX1 protein expression as a prognostic biomarker for solid tumors, we first investigated its correlation with clinicopathological features. Fifteen cohorts comprising 1,907 patients reported the relationship between PRDX1 expression and tumor size, and the pooled results indicated that high expression of PRDX1 was correlated with large tumor size (OR = 1.69, 95% CI: 1.07-2.68, *P* = 0.025, random effects). Moreover, PRDX1 overexpression had a significant association with advanced TNM stage (OR = 2.26, 95% CI: 1.24-4.13, *P* = 0.008, random effects) and poor tumor differentiation (OR = 0.59, 95% CI: 0.44-0.81, *P* = 0.001, fixed effects). However, PRDX1 expression was not related to age (OR = 0.99, 95% CI: 0.73-1.36, *P* = 0.966, random effects), gender (OR = 0.93, 95% CI: 0.74-1.17, *P* = 0.514, fixed effects), depth of invasion (OR = 1.11, 95% CI: 0.37-3.38, *P* = 0.854, random effects), lymph node metastasis (OR = 1.47, 95% CI: 0.93–2.34, *P* = 0.100, random effects), and distant metastasis (OR = 1.94, 95% CI: 0.84-4.47, *P* = 0.120, fixed effects). The results are shown in Table 2.

### 3.3. Impact of PRDX1 on Prognosis

**Figure 2:**
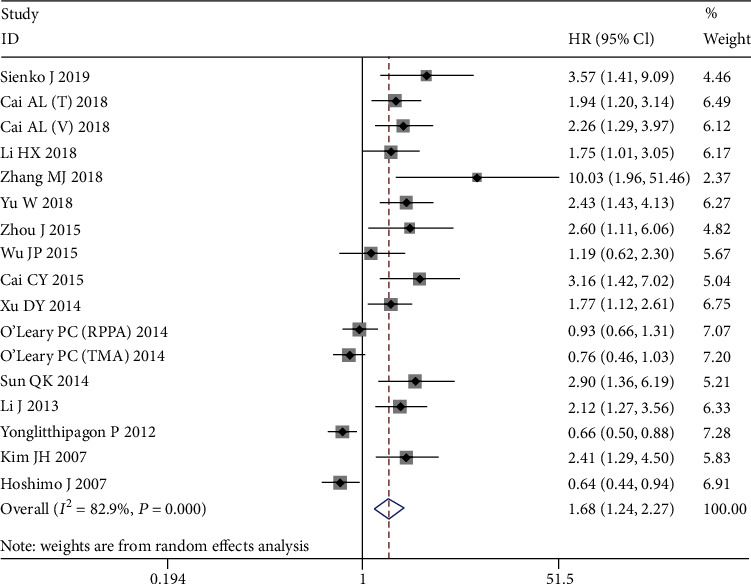
Forest plots of the overall outcomes for overall survival (OS). Hazard ratios (HRs) for each trial are represented by the squares, and the horizontal lines crossing the square stand for the 95% confidence intervals (CIs). The diamonds represent the estimated pooled effect of the overall outcome for OS in all solid tumors. All *P* values are two-sided.

**Figure 3:**
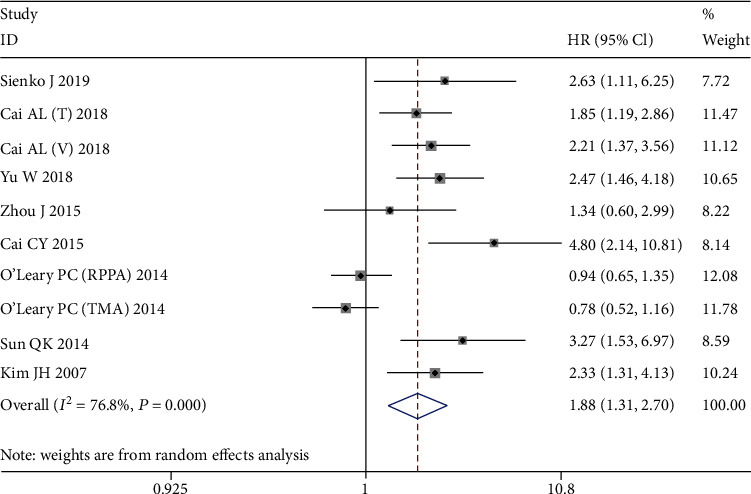
Forest plots of the overall outcomes for disease-free survival (DFS). Hazard ratios (HRs) for each trial are represented by the squares, and the horizontal lines crossing the square stand for the 95% confidence intervals (CIs). The diamonds represent the estimated pooled effect of the overall outcome for DFS in all solid tumors. All *P* values are two-sided.

The main results of this meta-analysis are listed in Table 3. Seventeen cohorts involving 2,858 patients were used to assess the relationship between PRDX1 protein expression and OS, and 10 cohorts used DFS as the endpoint. For OS, the random effects model was applied to pool the HRs and 95% CIs because of extreme heterogeneity (*I*^2^ = 82.9%, *P* < 0.001). The pooled results indicated that PRDX1 overexpression was significantly related to poor OS (HR = 1.68, 95% CI: 1.24-2.27, random effects, Figure 2). For DFS, the results showed the association of positive PRDX1 expression with unfavorable DFS (HR = 1.88, 95% CI: 1.31-2.70, random effects, Figure 3), and with significant heterogeneity (*I*^2^ = 76.8%, *P* < 0.001).

### 3.4. Subgroup and Metaregression Analyses for OS

Considering the significant heterogeneity among the studies, we conducted subgroup and meteregression analyses by focusing on study region, cancer type, TNM stage, sample size, and analysis method to explore sources of heterogeneity for OS (Table 3).

Subgroup analysis of the study region indicated that PRDX1 overexpression was significantly related to worse OS in eastern countries (HR = 1.79, 95% CI: 1.24-2.58, *P* = 0.002, random effects) but not in western countries (HR = 1.39, 95% CI: 0.78-2.48, *P* = 0.267, random effects). Subgroup analysis based on cancer type suggested that high PRDX1 expression predicted poor OS in patients with OC (HR = 4.60, 95% CI: 2.05-10.34, *P* < 0.001, fixed effects), OSC (HR = 2.07, 95% CI: 1.43-2.98, *P* < 0.001, fixed effects), GC (HR = 2.00, 95% CI: 1.44-2.79, *P* < 0.001, fixed effects), and others (HR = 2.27, 95% CI: 1.72-3.00, *P* < 0.001, fixed effects). Nevertheless, no significant relationship was observed between PRDX1 expression and OS in patients with CCA (HR = 1.23, 95% CI: 0.32-4.69, *P* = 0.757, random effects), ESCC (HR = 0.82, 95% CI: 0.45-1.50, *P* = 0.521, random effects), and BC (HR = 0.83, 95% CI: 0.66-1.04, *P* = 0.111, fixed effects). With regard to TNM stage, the results showed the predictive role of PRDX1 positive expression on unfavorable OS in patients with cancer of stages I-IV (HR = 2.03, 95% CI: 1.38-2.98, *P* < 0.757, random effects), and I (HR = 2.41, 95% CI: 1.29-4.50, *P* = 0.006, random effects), but not in those with stages I-III (HR = 1.27, 95% CI: 0.77-2.10, *P* = 0.341, random effects), and none reported (HR = 0.66, 95% CI: 0.50-0.88, *P* = 0.004, random effects). When the cohorts were grouped by sample size, high PRDX1 expression was associated with worse OS for small samples but better OS for large sample sizes. Finally, the subgroup analysis suggested the close association of PRDX1 overexpression with worse OS in the multivariate analysis (HR = 1.28, 95% CI: 1.28-2.58, *P* < 0.001, random effects) but not in the univariate analysis (HR = 1.24, 95% CI: 0.61-2.53, *P* = 0.555, random effects). Further metaregression analysis revealed that sample size (*P* = 0.010) and analysis method (*P* = 0.034) may be important factors for heterogeneity.

### 3.5. Sensitivity Analysis and Publication Bias

**Figure 4:**
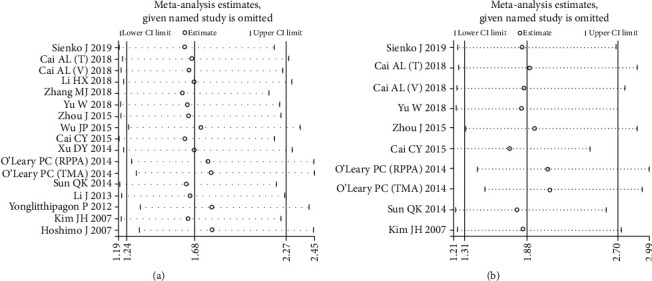
Effects of individual studies on pooled hazard ratios (HRs) for PRDX1 and survival in solid tumors. (a) Result of sensitivity analysis for pooled OS estimation. (b) Result of sensitivity analysis for pooled DFS estimation.

Sensitivity analysis was performed to assess the robustness of our results by sequentially omitting each individual cohort. No significant change was detected in the combined HR estimates of OS (Figure 4(a)) and DFS (Figure 4(b)). This finding indicated that our results were stable and reliable.

**Figure 5:**
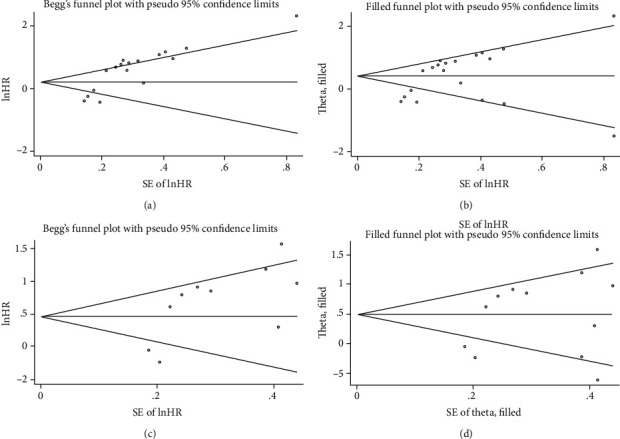
Begg's funnel plots for assessment of potential publication bias in studies of PRDX1 in patients with solid tumor. Each study represented by one circle. The horizontal line represented the pooled effect estimate. (a) Funnel plot of publication bias for overall survival. (b) Funnel plot adjusted with trim-and-fill methods for overall survival. (c) Funnel plot of publication bias for disease-free survival. (d) Funnel plot adjusted with trim-and-fill methods for studies reporting disease-free survival.

For OS, significant bias was found by Begg's test (*P* = 0.002) and Egger's test (*P* < 0.001). Furthermore, the funnel plot visually showed an apparent asymmetry (Figure 5(a)). The trim-and-fill analysis indicated that three unpublished studies were needed to neutralize the potential bias (Figure 5(b)). The combined results were slightly changed but remained significant (HR = 1.48, 95% CI: 1.11–1.97, random effects). Thus, potential publication bias exerted minimal effect on the pooled results. For DFS, a significant publication bias was confirmed by Egger's test (*P* = 0.023) but not by Begg's test (*P* = 0.107), which was also revealed by the asymmetrical funnel plot (Figure 5(c)). According to the trim-and-fill analysis, two nonpublished articles were needed to balance the funnel plot (5D), and the pooled outcomes remained significant (HR = 1.62; 95% CI: 1.15–2.30, random effects). Therefore, the results were considered reliable.

## 4. Discussion

PRDX1 is a multifunctional protein that acts as a hydrogen peroxide scavenger, molecular chaperone, and immune modulator [29]. The relationship between PRDX1 expression level and prognosis of patients with solid tumors has been studied; however, the prognostic value of PRDX1 remains highly ambiguous. Therefore, we performed this meta-analysis to critically assess the prognostic significance of PRDX1 expression.

In our meta-analysis, we pooled 17 cohort studies involving 2,858 patients with solid cancer. Our overall pooled results indicated that high PRDX1 expression could predict poor OS (HR = 1.68, 95% CI: 1.24-2.27, *P* = 0.001) and DFS (HR = 1.88, 95% CI: 1.31-2.70, *P* = 0.001). Hence, high PRDX1 expression could indicate poor prognosis in solid cancers. However, the combined results may be challenged because of significant heterogeneity. To explore the source of heterogeneity for OS, we conducted subgroup and metaregression analyses according to study region, cancer type, TNM stage, sample size, and analysis method. Sample size and analysis method may significantly explain the heterogeneity for the combined HR of OS, and the heterogeneity mainly originated from the studies of O'Leary et al. [29], Yonglitthipagon et al. [25], and Hoshino et al. [27]. Furthermore, sensitivity analysis and publication bias assessment were conducted to verify the stability and reliability of the combined results for OS and DFS. The sensitivity analysis suggested the absence of point estimate of the omitted individual dataset outside the 95% CI of the combined analyses. Despite the existence of significant publication bias, the trim-and-fill analysis confirmed that it had no strong effect on the pooled results of the meta-analysis.

To further verify the prognostic effect of PRDX1 on malignant tumors, we analyzed the correlation between PRDX1 expression and clinicopathological features that might affect survival outcomes. According to the pooled results, high PRDX1 expression was associated with large tumor size, advanced TNM stage, and poor tumor differentiation. No statistically significant correlations were found for age, gender, depth of invasion, lymph node metastasis, and distant metastasis. PRDX1 overexpression was related to poor prognosis and tumor invasiveness in solid cancers. The possible reasons for these results are as follows:

First, as an endogenous product of aerobic respiration in all metazoan organisms, ROS is involved not only in tumor metastasis but also in tumorigenesis [33, 34]. PRDX1 directly effects tumor suppression by eliminating ROS and preventing oxidative damage to DNA [35]. However, PRDX1 and other peroxidases have been reported to be overexpressed in some human solid tumors, thereby suggesting that tumors generated by other mechanisms may benefit from increased peroxidases [35]. PRDX1 cooperates with thioredoxin in inhibiting ROS-induced tumor cell apoptosis and promoting tumor cell survival by involving different types of kinases and enzymes, such as apoptosis signal-regulating kinase 1, p66^Shc^, and glutathione S-transferase pi (GSTpi)/c-Jun NH2-terminal kinase (JNK) [19, 36]. In addition, PRDX1 is considered a physiological inhibitor of c-Abl tyrosine kinase by binding to the SH-3 domain of c-Abl. c-Abl is an upstream effector molecule of the JNK and p38 mitogen-activated protein kinase pathway and plays a key role in oxidative stress-induced cell death [37]. Therefore, PRDX1 may inhibit stress-induced cell death through ROS-dependent signaling pathways [19]. The role of oxidative stress in ER-positive breast cancer may differ from those in other types of tumors. Physiological estrogen concentration can induce ROS production, which is the main carcinogenesis mechanism related to estrogen; as such, the ROS scavenging system is predicted to play an important role. Therefore, PRDX1 may be an independent predictor of improved prognosis of ER-positive breast cancer (which differs from other cancers) [29, 38]. Moreover, any possible PRDX1 inhibitors should be carefully explored in ER-positive breast cancer because they may transform tumor cells into a more aggressive phenotype with poor prognosis. This situation is also the main source of heterogeneity in this meta-analysis. The possible reasons of heterogeneity caused by two articles in this work were as follows: the HR in one study was not adjusted for confounding factors, and patients in the other article were infected with liver fluke.

Second, previous studies revealed that PRDX1 excessive expression enhances epithelial-mesenchymal transition (EMT) by inducing transforming growth factor beta 1; EMT is regarded as a critical process in tumor invasion and metastasis [39]. During EMT, malignant tumor cells lose their own epithelial properties and acquire the characteristics of mesenchymal cells, thereby causing the dissociation of tumor cells from primary carcinomas and promoting their subsequent migration and dissemination to distant sites [22, 40]. EMT is also an important event in the tumor microenvironment, and various components of such microenvironment provide suitable conditions for EMT [28, 41].

Third, the extracellular matrix (ECM) is a component of the tumor microenvironment, and its degradation is essential for tumor cells to escape from primary lesions. The matrix metalloproteinase (MMP) family can degrade the major components of the ECM and plays an important role in promoting tumor invasion and metastasis [42]. Among MMP types, MMP2 and MMP9 promote the invasion and metastasis of tumor cells by degrading type IV collagen, a main component of the ECM [21]. A study of 180 patients with CRC showed that MMP2 and MMP9 overexpression was associated with poor prognosis [43]. Moreover, MMP could be activated by PRDX1 to cause tumor cell metastasis [21].

Fourth, the invasion and metastasis of tumor cells are a complex series of pathophysiological processes accompanied by a complex regulatory network involving multiple molecules. In addition to the signaling pathways mentioned above, those related to PRDX1 are involved in the p53 signaling pathway, Akt/mTOR signaling pathway, PI3K/Akt signaling pathway, pathways in cancer, FoxO signaling pathway, cell cycle, and ubiquitin-mediated proteolysis [8, 20, 44].

This meta-analysis presents certain limitations, and the results should be interpreted with caution. To begin with, the cut-off value of positive and negative PRDX1 expression was inconsistent among different studies, leading to bias in the results. Second, several individual HRs could not be directly achieved from the published data, resulting in some errors. Third, studies with positive results were more likely to be published than those with negative results. This phenomenon may lead to publication bias and exaggerate the overall results. Fourth, only published articles in English and Chinese were enrolled. Fifth, all included cohort studies were retrospective. Prospective studies are needed to assess the prognostic value of PRDX1 expression in patients with solid tumors. Finally, although we used the random effects model for the analysis, a considerable heterogeneity persisted in our study.

## 5. Conclusion

Despite the limitations of this meta-analysis and the heterogeneity of the included studies, our results suggest that high PRDX1 expression is significantly associated with poor prognosis and may serve as a new biomarker for monitoring the development and progression of tumors. Hence, PRDX1 could be a new target for antitumor therapy. Large-scale prospective and standard investigations should be conducted in the future to confirm our results.

## Figures and Tables

**Table 1:** Main characteristics of the eligible studies.

Study	Study region	Duration	Cancer type	TNM stage	Follow-up (months)	Number	Technology	PRDX1-high (%)	Analysis method	HR (95% CI)	Language	Quality
Sieńko J 2019	Poland	2003-2010	OC	I-IV	Mean 60.23	55	IHC	11 (20.0%)	Multivariate	OS: 3.571 (1.408-9.091) DFS: 2.632 (1.111-6.250)	English	8
Cai AL (T) 2018	China	2007-2010	OSC	I-III	Mean 63	110	IHC	66 (60.0%)	Multivariate	OS: 1.938 (1.197-3.138) DFS: 1.845 (1.191-2.856)	English	7
Cai AL (V) 2018	China	2007-2010	OSC	I-III	Mean 63	90	IHC	59 (65.6%)	Multivariate	OS: 2.261 (1.288-3.970) DFS: 2.213 (1.374-3.564)	English	7
Li HX 2018	China	2009-2012	CRC	I-IV	NR	60	IHC	42 (70.0%)	Univariate	OS: 1.754 (1.010-3.046)	English	6
Zheng MJ 2018	China	2008-2012	OC	I-IV	Up to 2017.9	101	IHC	17 (16.8%)	Multivariate	OS: 10.033 (1.956-51.462)	English	8
Yu W 2018	China	2008-2012	GC	I-IV	NR	189	IHC	127 (67.2%)	Multivariate	OS: 2.433 (1.429-4.132) DFS: 2.469 (1.456-4.184)	English	7
Zhou J 2015	China	2004-2008	CCA	I-IV	Median 16	88	IHC	53 (60.2%)	Multivariate	OS: 2.597 (1.114-6.061) DFS: 1.344 (0.603-2.994)	English	8
Wu JP 2015	China	2005-2014	ESCC	I-IV	NR	144	IHC	96 (66.7%)	Multivariate	OS: 1.192 (0.616-2.304)	Chinese	7
Cai CY 2015	China	2008-2011	PC	I-IV	Median 13.5	86	IHC	64 (74.4%)	Multivariate	OS: 3.162 (1.425-7.018) DFS: 4.805 (2.136-10.812)	English	8
Xu DY 2014	China	2001-2003	GC	I-IV	Median 30	120	IHC	70 (58.3%)	Univariate	OS: 1.769 (1.120-2.609)	Chinese	6
O'Leary PC (RPPA) 2014	USA, Spain, Canada	NR	BC	I-III	NR	712	RPPA	356 (50.0%)	Multivariate	OS:0.93 (0.66-1.31) DFS: 0.94 (0.65-1.35)	English	7
O'Leary PC (TMA) 2014	Sweden	1987-1992	BC	I-III	Median 11	442	IHC	221 (50.0%)	Multivariate	OS: 0.76 (0.56-1.03) DFS: 0.78 (0.52-1.16)	English	8
Sun QK 2014	China	2006-2009	HCC	I-IV	Median 24	76	IHC	56 (73.7%)	Multivariate	OS: 2.897 (1.355-6.194) DFS: 3.268(1.532-6.971)	English	8
Li J 2013	China	2001-2009	GBC	I-IV	NR	80	IHC	42 (52.5%)	Multivariate	OS: 2.123 (1.266-3.562)	English	7
Yonglitthipagon P 2012	Thailand	NR	CCA	NR	NR	301	IHC	102 (33.9%)	Multivariate	OS: 0.662 (0.498-0.877)	English	7
Kim JH 2007	USA	1993-2002	NSCLC	I	Median 70	90	IHC	60 (66.7%)	Multivariate	OS: 2.409 (1.290-4.498) DFS: 2.327 (1.312-4.128)	English	8
Hoshino I 2007	Japan	NR	ESCC	I-IV	NR	114	IHC	41 (36.0%)	Univariate	OS: 0.640 (0.436-0.937)	English	6

OC: ovarian cancer; OSC: osteosarcoma; CRC: colorectal cancer; GC: gastric cancer; CCA: cholangiocarcinoma; ESCC: esophageal squamous cell carcinoma; PC: pancreatic cancer; BC: breast cancer; HCC: hepatocellular carcinoma; GBC: gallbladder cancer; NSCLC: non-small cell lung cancer; OS overall survival; DFS: disease-free survival; HR: hazard ratio; CI: confidence interval; NR: none reported; T: Training cohort; V: Validation cohort; RPPA: reverse phase protein array; TMA: microarray; IHC: immunohistochemistry.

**Table 2:** Meta-analysis of PRDX-1 and clinicopathological features in solid tumors patients.

Categories	Trials (patients)	OR (95% CI)	*I* ^2^(%)	*P*_*h*_	*Z*	*P*
Age (young vs. old)	15 (2564)	0.99 (0.73-1.36)	62.4%	0.001	0.04	0.966
Gender (male vs. female)	13 (1543)	0.93 (0.74-1.17)^F^	21.5%	0.226	0.65	0.514
Tumor size (small vs. large)	12 (1907)	1.69 (1.07-2.68)	77.8%	<0.001	2.24	0.025
TNM stage (I+II vs. III+IV)	14 (2110)	2.26 (1.24-4.13)	87.3%	<0.001	2.65	0.008
Depth of invasion (T1+T2 vs. T3+T4)	4 (567)	1.11 (0.37-3.38)	87.1%	<0.001	0.18	0.854
Lymph node metastasis (negative vs. positive)	10 (1847)	1.47 (0.93-2.34)	76.9%	<0.001	1.64	0.100
Distant metastasis (negative vs. positive)	2 (174)	1.94 (0.84-4.47)^F^	26.9%	0.242	1.55	0.120
Degree of differentiation (poor/not vs. well/moderate)	7 (808)	0.59 (0.44-0.81)^F^	21.2%	0.268	3.33	0.001

All pooled ORs were calculated from random-effect model except for cells marked with (fixed^F^). *P*_*h*_ denotes *P* value for heterogeneity based on *Q* test; *P* denotes *P* value for statistical significance based on *Z* test. OR, odds ratio; CI, confidence interval.

**Table 3:** Summary of the meta-analysis results.

Categories	Trials (patients)	HR (95% CI)	*I* ^2^(%)	*P*_*h*_	*Z*	*P*	*P*_*m*_
OS (all)	17 (2858)	1.68 (1.24-2.27)^R^	82.9%	<0.001	3.35	0.001	
Study region							0.474
Eastern countries	13 (1559)	1.79 (1.24-2.58)^R^	83.1%	<0.001	3.11	0.002	
Western countries	4 (1299)	1.39 (0.78-2.48)^R^	83.4%	<0.001	1.11	0.267	
Cancer type							0.641
OC	2 (156)	4.60 (2.05-10.34)^F^	13.6%	0.282	3.69	<0.001	
OSC	2 (200)	2.07 (1.43-2.98)^F^	0.0%	0.683	3.89	<0.001	
GC	2 (309)	2.00 (1.44-2.79)^F^	0.0%	0.357	4.11	<0.001	
CCA	2 (389)	1.23 (0.32-4.69)^R^	88.9%	0.003	0.31	0.757	
ESCC	2 (258)	0.82 (0.45-1.50)^R^	60.9%	0.110	0.64	0.521	
BC	2 (1154)	0.83 (0.66-1.04)^F^	0.0%	0.388	1.60	0.111	
Others	5 (392)	2.27 (1.72-3.00)^F^	0.0%	0.736	5.77	<0.001	
TNM stage							0.601
I-IV	11 (1113)	2.03 (1.38-2.98)^R^	75.4%	0.001	3.62	<0.001	
I-III	4 (1354)	1.27 (0.77-2.10)^R^	83.4%	<0.001	0.95	0.341	
I	1 (90)	2.41 (1.29-4.50)^R^	—	—	2.76	0.006	
NR	1 (301)	0.66 (0.50-0.88)^R^	—	—	2.86	0.004	
Sample size							0.010
≥300	3 (1455)	0.76 (0.64-0.91)^F^	11.0%	0.325	8.96	<0.001	
<300	13 (1403)	2.03 (1.51-2.74)^R^	69.9%	0.001	4.67	<0.001	
Analysis method							0.034
Multivariate	14 (2564)	1.82 (1.28-2.58)^R^	83.4%	<0.001	3.34	0.001	
Univariate	3 (294)	1.24 (0.61-2.53)^R^	86.9%	<0.001	0.59	0.555	
DFS (all)	10 (1908)	1.88 (1.31-2.70)^R^	76.8%	<0.001	3.43	0.001	

OC: ovarian cancer; OSC: osteosarcoma; GC: gastric cancer; CCA: cholangiocarcinoma; ESCC: esophageal squamous cell carcinoma; BC: breast cancer; others including colorectal cancer, pancreatic cancer, hepatocellular carcinoma, gallbladder cancer, and non-small cell lung cancer; F: fixed effects; R: random effects; NR: none reported; OS overall survival; DFS: disease-free survival; HR: hazard ratio; CI: confidence interval; *P*_*z*_: *P* value for statistical significance based on *Z* test; *P*_*h*_: *P* value for heterogeneity based on *Q* test; *P*_*m*_: *P* value for statistical outcome based on multivariate metaregression analysis.

## Data Availability

The data supporting this meta-analysis are from previously reported studies and datasets, which have been cited. The processed data are available within the article.
